# Altered Toll-Like Receptor-4 Response to Lipopolysaccharides in Infants Exposed to HIV-1 and Its Preventive Therapy

**DOI:** 10.3389/fimmu.2018.00222

**Published:** 2018-02-14

**Authors:** Anicet Christel Maloupazoa Siawaya, Ofilia Mvoundza Ndjindji, Eliane Kuissi Kamgaing, Amandine Mveang-Nzoghe, Chérone Nancy Mbani Mpega, Marielle Leboueny, Roselyne Kengue Boussougou, Armel Mintsa Ndong, Paulin N. Essone, Joel Fleury Djoba Siawaya

**Affiliations:** ^1^Centre Hospitalier Universitaire Mère-Enfant Fondation Jeanne Ebori (CHUMEFJE), Libreville, Gabon; ^2^Unités de Recherche et de Diagnostics Spécialisés, Laboratoire National de Santé Publique à Libreville (LNSP), Libreville, Gabon; ^3^Département de Pédiatrie, Université des Sciences de la Santé d’Owendo (USS), Owendo, Gabon; ^4^Service de Néonatologie, Centre Hospitalier Universitaire de Libreville (CHUL), Libreville, Gabon; ^5^Département de Chimie, Faculté des Sciences, Université des sciences et techniques de Masuku, Franceville, Gabon; ^6^Unité de Virologie, Laboratoire National de Santé Publique à Libreville (LNSP), Libreville, Gabon; ^7^Centre de Recherche Médicales de Lambaréné, Lambaréné, Gabon; ^8^Institut für Tropenmedizin, Universitätsklinikum Tübingen, Tübingen, Germany

**Keywords:** infants, HIV-1, toll-like receptor-4, complement component-3, C-reactive protein

## Abstract

Pathogen sensing and recognition through pattern recognition receptors, and subsequent production of pro-inflammatory cytokines, is the cornerstone of the innate immune system. Despite the fact that HIV-exposed uninfected (HEU) infants are prone to serious bacterial infections, no study has focused on the functionality of their bacteria recognition system. This is the first study to investigate baseline levels of three critically important immune response molecules in this population: complement component (C)-3, toll-like receptor (TLR)-4, and C-reactive protein (CRP). We enrolled 16 HEU and 6 HIV-unexposed (HU) infants. TLR4 function was investigated by stimulating whole blood with increasing concentrations of TLR4-agonist ultrapure lipopolysaccharides. TLR4/TLR4-agonist dose response were assessed by measuring IL-6 secretion. Complement C3 and CRP were measured by photo spectrometry. Data showed no significant differences in baseline concentration of CRP between HEU and HU infants. Complement C3 was significantly higher in HEU infants than HU infants. TLR4 anergy was observed in 7 of 12 HEU infants, whereas the rest of HEU infants (*n* = 4) and the control HU infants tested (*n* = 3) showed responsive TLR4. None of the HEU infants investigated in this study had severe infections in the year after their birth. In conclusion, TLR4 anergy can occur in HEU infants without necessarily translating to increased vulnerability to infectious diseases.

## Introduction

Progress in the prevention of mother-to-child transmission of HIV has led to a significant reduction of mother-to-child HIV transmission rate, increasing the population of HIV-exposed uninfected (HEU) infants (1–3). Indeed, HEU infants are a growing population with over a million infants born every year from HIV-infected mothers (2). Studies have shown that HEU infants are more vulnerable to diseases than infants born from HIV free mothers (4–8). The global efforts to understand HEU infants’ increased susceptibility to infections during the first months of their life has shed some light on the immune determinants of their susceptibility. We recently showed impaired oxidative burst in neutrophils from a number of HEU infants (9). Others have reported impaired humoral response (10, 11), altered chemokine receptor expression by CD4^+^ T cells (12), altered natural killer cell function, and reduced thymic output of naive CD4^+^ cells (13).

The innate immune sensing and recognition of microbial elements is critical for the host defense against infection (14). Pattern recognition receptors such as toll-like receptors (TLRs) that detect pathogen-associated molecular patterns (PAMPs) are the hallmark of pathogen sensing, which triggers both innate and adaptive immune response (14). TLRs are type 1 transmembrane receptors. So far 10 functional TLRs have been described in humans including TLRs 1, 2, 4, 5, 6, 7, 8, 9, and 10, each with distinct functions. TLR4 is activated by lipopolysaccharides (LPS) found in Gram-negative bacteria (e.g., *Klebsiella pneumoniae, Haemophilus influenza*, etc.) (15, 16), which are a significant cause of morbidity and mortality among infants (17). TLR4 plays a central role in defense against Gram-negative bacteria; however, mutations on the TLR4 gene or TLR4-deficient mice have been linked to hypo-responsiveness to LPS and high susceptibility to Gram-negative bacterial infections (15, 18, 19).

In addition to TLR 4, complement component (C)-3 and C-reactive protein (CRP) are also important in innate immune recognition and killing of invading pathogens. The complement C3 is the most abundant complement component in plasma, plays a key role in the activation of complement system, and is required for both classical and alternative complement activation pathways (20). Proteolytic cleavage of the C3 α-chain leads to C3a (a key inflammatory mediator) and C3b (a major opsonin) (20). CRP plays a major role in host defense against infection and in inflammatory processes (21, 22); it is a pro-inflammatory mediator, a pathogen surveillance molecule, an opsonin for diverse pathogens, and an activator of complement that provides early defense against invading pathogens (23).

Because of the critical role of these three molecules, we investigated the function of TLR4 and assessed the baseline levels of complement C3 and CRP in HEU infants. Our results contribute to a global effort to understand the immunity of this infant population. Understanding HEU infants’ immunity is essential to the development of new health-care policies (therapeutic, vaccines, etc.) to combat infectious agents.

## Materials and Methods

### Subjects

The present prospective study included 16 HEU and 6 HIV-unexposed (HU) infants from the National Laboratory of Public Health in Libreville (Gabon). Whole blood was collected in 5 ml EDTA tubes and transported to the laboratory for processing. The national laboratory of public health ethical committee approved this study. Written informed consent was obtained from parents for individual participants. Mothers’ and infants’ information on preventive treatment, breastfeeding, and time of antiretroviral therapy initiation were also collected. All mothers came from modest background.

HIV perinatal infection (RT-PCR, NucliSENS, Biomerieux, France) was checked from each HEU infant in peripheral blood at 1.5, 3, 6, and 9 months after birth as part of their scheduled check up in the mother-to-child transmission prevention programs reference laboratory. An additional sample was taken to determine anti-HIV-1 antibodies seroconversion by ELISA at 18 months old. During each scheduled visit, infants’ medical records were checked and mothers were asked if infants had experienced any severe infection or if they were hospitalized. All HIV-positive infants and infants with any illness or infection at the time of recruitment were excluded from the study.

### Baseline Complement C3 and CRP Levels (Circulating Levels of Complement C3 and CRP in Absence of Any Stimulus)

Circulating levels of complement C3 and CRP in HEU infants (*N* = 11) and HU infants (*N* = 6) were assessed using MD Pacific kits (C3: Ref MD015.SL and CRP: Ref MD021.SL) and protein analyzer (MD Pacific Biotechnology, Tianjin, China). The protocol was performed according to the manufacturer’s instructions. Briefly, whole blood samples were centrifuged before plasma collection. 20 µl of the collected plasma was diluted 1:10 in sample diluent and read on the MD Pacific analyzer to measure the quantity of complement C3 and CRP.

### TLR4 Activation Profile Assay

A volume of 200 µl of freshly drawn whole blood was directly transferred into TLR4 test strips pre-coated with a TLR4 agonist at different concentrations (ultrapure LPS at: 0, 0.001, 0.01, 0.1, 1, 10, 100, and 1,000 ng) (Ref tlrs-tlr4, Invivogen, San Diego, CA, USA). All study participants with less than 1.6 ml of whole blood were excluded from this experiment. The TLR4 test strips containing blood samples from infants were incubated overnight at 37°C in a humidified 5% CO_2_ incubator. The following day, blood supernatants were collected and stored at −40°C for 18 months. We were able to harvest enough plasma from 12 HEU and 3 HU infants after whole blood stimulation with LPS.

### IL-6 Expression

Frozen whole blood supernatants from the TLR4/TLR4-agonist stimulation assay were thawed and diluted 1/10 in assay diluent. IL-6 concentration was measured using Elecsys cartridge (Ref 05109442190, Roche Diagnostics, Germany) on the Cobas e411 analyzer (Roche Diagnostics, Germany). The test measuring range was 1.5–5.000 pg/ml. Samples with an IL-6 concentration above the test limit of 5,000 pg/ml were further diluted (in assay diluent) and reanalyzed.

### Statistical Analyses

The levels of complement C3 and CRP between the two groups of infants were analyzed using the Mann–Whitney *U*-test with an alpha value of 0.05. For each infant, a TLR4/TLR4-agonist dose–response curve was generated and analyzed by linear regression. All statistical analyses were done on GraphPad Prism 6 (GraphPad Software, Inc., USA).

## Results

Information on mothers’ and infants’ preventive treatment, breastfeeding, and time of antiretroviral therapy initiation are presented in Table 1. All HIV-positive mothers followed the mother-to-child preventive therapy. All infants except one followed the nevirapine and co-trimoxazole preventive therapy. 92% of infants were not breastfed.

**Table 1:** Participants anthropometric and clinical information.

Infant ID	Age (in weeks)^a^	Gender	Infants preventive Thx	Mother preventive Thx	Breast feeding	Mother start of ART			Hospitalization due to severe infection in the year following birth
						Before pregnancy	During pregnancy	Therapy	
HEU1	8	Female	Nevirapine/co-trimoxazole	YES	NO	YES	NO	TDF-3TC-EFV	NO

HEU2	7	Male	Nevirapine/co-trimoxazole	YES	NO	NO	YES	TDF-FTC-EFV	Data not available

HEU3	6	Female	Zidovidine/co-trimoxazole	YES	NO	YES	NO	Data not available	NO

HEU4	6	Female	Nevirapine/co-trimoxazole	YES	NO	NO	YES at 5 month	TDF-3TC-EFV	NO

HEU5	6	Male	Nevirapine/co-trimoxazole	YES	NO	YES	NO	Data not available	NO

HEU6	6	Female	Nevirapine/co-trimoxazole	YES	NO	NO	YES at 5 month (but interrupted)	Data not available	Data not available

HEU7	7	Male	Excluded (HIV-infected)						

HEU8	7	Male	Nevirapine/co-trimoxazole	YES	NO	YES	NO	TDF-3TC-EFV	NO

HEU9	8	Male	Nevirapine/co-trimoxazole	YES	YES	NO	YES at 4 month (but interrupted)	Data not available	NO

HEU10	8	Male	Nevirapine	YES	YES	YES	YES at 4 month (but interrupted)	TDF-FTC-EFV	Data not available

HEU11	6	Female	Nevirapine/co-trimoxazole	YES	NO	YES	NO	TDF-FTC-EFV	NO

HEU12	6	Female	Nevirapine/co-trimoxazole	YES	NO	NO	YES at 4 month		Data not available

HEU13	6	Male	Nevirapine/co-trimoxazole	YES	NO	YES	NO	TDF-3TC-EFV	NO

HEU14	8	Female	Nevirapine/co-trimoxazole	YES	NO	YES	Interrupted	Data not available	Data not available

HEU15	8	Male	NO	YES	Data not available	YES	NO	Data not available	NO

HEU16	8	Female	Nevirapine/co-trimoxazole	YES	Data not available	NO	YES	Data not available	Data not available

HU1	6	Male	NA	NA	YES	NA	NA	NA	Data not available

HU2	6	Male	NA	NA	YES	NA	NA	NA	Data not available

HU3	6	Female	NA	NA	YES	NA	NA	NA	Data not available

HU4	7	Female	NA	NA	YES	NA	NA	NA	Data not available

HU5	8	Female	NA	NA	YES	NA	NA	NA	Data not available

HU6	8	Male	NA	NA	YES	NA	NA	NA	Data not available

*ART, antiretroviral therapy, HEU, HIV-exposed uninfected, HU, HIV unexposed; Thx, therapy; 3TC, lamivudine; EFV, efavirenz; FTC, emtricitabine; TDF, tenofovir disoproxil fumarate; Data not available, mother could not provide us with the name of the ARV she was on; NA, not applicable*.

*^a^Enrollment and blood collection age*.

### High Level of Complement C3 in HEU Infants

**Figure 1:**
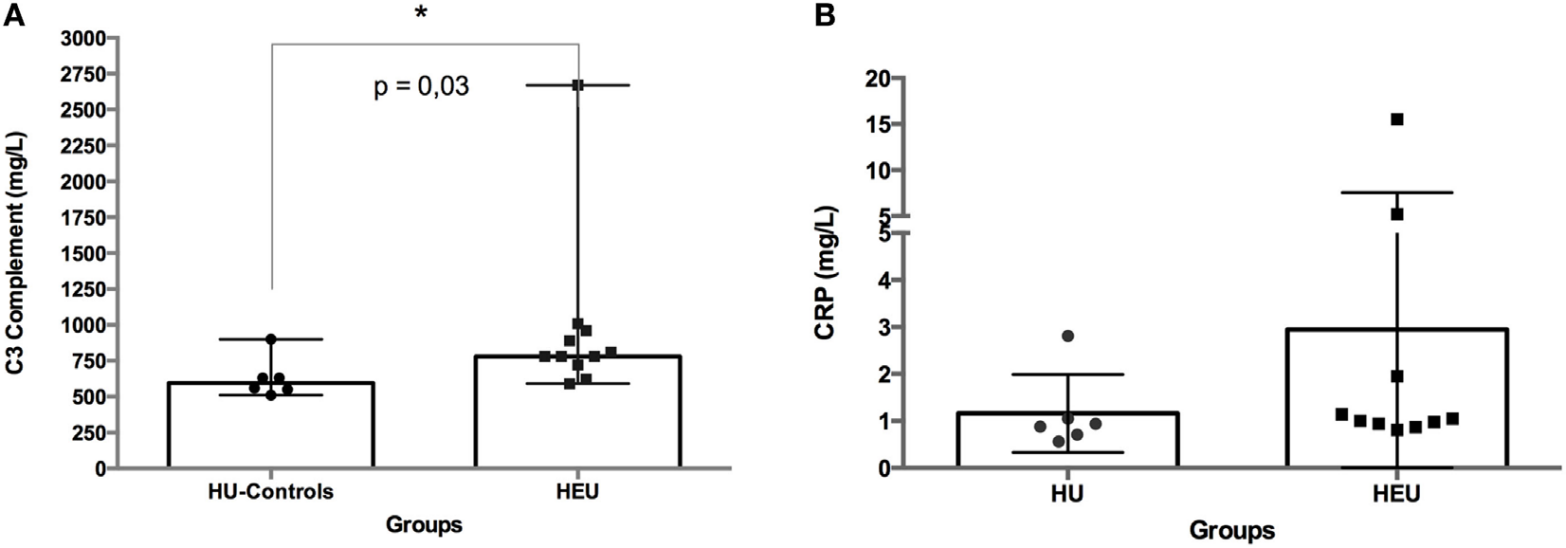
Circulating levels of C3 complement **(A)** and C-reactive protein (CRP) **(B)** in whole blood from HIV-exposed and HIV-unexposed (HU) infants. C3 complement and CRP were measured in infants’ serum samples. The difference between HIV-exposed and HU infants was analyzed using the Mann–Whitney *U*-test. The differences were considered significant for a *p*-value <0.05. In the figures, bars indicate medians and ranges.

To assess the baseline inflammatory status of infants (HEU: *N* = 11; HU: *N* = 6), we investigated the circulating levels of complement C3 and CRP. The level of complement C3 was significantly higher in HEU infants compared with HU infants (median of 780 vs. 595; *p* = 0.03). No significant difference was observed between infants’ groups for CRP (Figure 1).

### Response to TLR4-Agonist

**Figure 2:**
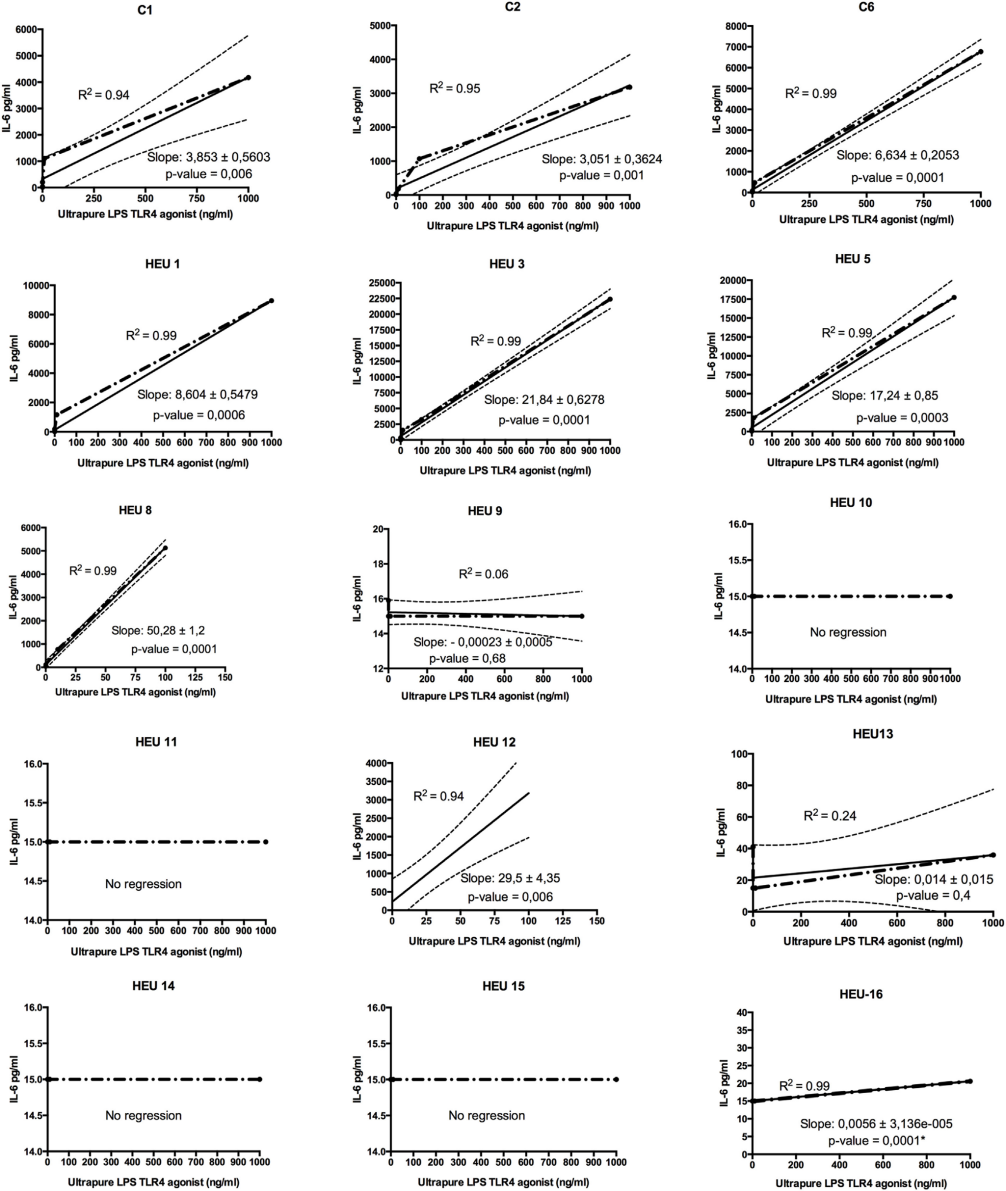
Regression analysis of toll-like receptor (TLR)-4/ultrapure lipopolysaccharides (LPS) dose–response of HIV-exposed uninfected (HEU) infants and HIV-unexposed infants (C). Whole blood from individual participant was stimulated at increasing concentration of LPS (from 0, 0.001 to 1,000 ng), and IL-6 was measured. The slope indicator measures the rise over the run for a linear regression. The *p*-value simply tests whether the slope is exactly 0 or ≠0. The coefficient of determination means that *R*^2^ × 100% of the variation in IL-6 can be explained by linear relationship with TLR4-agonist stimulation. (*) Only the one point deviated significantly from 0 (corresponding to the top LPS stimulation).

HIV-exposed uninfected infants showed two distinct TLR4 response profiles when stimulated for 24 h with ultrapure LPS (TLR4-agonist) (Figure 2). More than half of HEU infants (7 of 12) did not respond to LPS stimulation as shown by their IL-6 secretion profiles. Despite stimulation with increasing concentration of TLR4 agonist (0.0001–1,000 ng/ml), in the hyporesponsive group, IL-6 secretions did not exceed 41 pg/ml even for the top agonist concentration. The remaining HEU and HU infants showed enhanced IL-6 secretions to TLR4-agonist stimulations (Figure 2). We observed a positive correlation between IL-6 secretion and LPS concentration. This correlation was similar in all LPS responders independently of their HIV exposure status.

Linear regression analysis of the TLR4 dose–response assay confirmed that selected HEU infants had hyporesponsive TLR4. Indeed, six healthy infants born from HIV-positive women (HEU 9, 10, 11, 13, 14, and 15) showed no significant or no deviation of their dose–response slopes. Further, one HEU infant (HEU-16) showed a dose–response slope deviating significantly from zero. However, the slope, which represents the rise across an increasing concentration of TLR4-agonist, was very weak (0.0056), indicating a very weak response to TLR4-agonist. The remaining five HEU infants showed a linearity between TLR4-agonist concentration and IL-6 secretion.

## Discussion

There is evidence that *in utero* exposure to HIV and antiretroviral therapy (ART) have knock-on health effects on infants, including an increased susceptibility to bacterial infection (4–7, 24–27). Research on the etiology of HEU infants’ susceptibility has highlighted a number of immune abnormalities in these infants (9, 10, 25, 28–30). We believe that the benefits of ART in preventing HIV vertical transmission and improving mother and child health are undeniable (31). Although these benefits certainly outweigh the potential adverse effects of ART exposure to infants, these adverse effects should not be neglected, as they can be detrimental to infants’ health (25).

In this study, half of the mothers started therapy during pregnancy, two of them interrupted their treatment, and 92% of infants were not breastfed. This is not surprising as, in Gabon, despite free antenatal care, early access, and adherence to components of mother to child, HIV transmission preventive care remains unsatisfactory (3). Therefore, mothers are often advised not to breastfeed if they are not fully compliant to the ARV therapy.

Complement C3, plays a key role in both classical and alternative complement activation pathways (20). Here, we showed that HEU infants had significantly higher concentrations of complement C3 compared to the control group of HU infants. Hemolytic activity and components of the complement system (32) are low in newborn; therefore, the low levels of complement C3 in our group of HU infants are not surprising. Viral particles including HIV are known to induce the production and activate complement C3 (33–35). Also, studies showed that fetal exposure to HIV and ART skews newborns infant’s physiological immune responses toward inflammation (36). Therefore, HEU infants *in utero* exposure to HIV particles and pro-inflammatory environment (37) could explain the higher level of complement C3 found in HEU infants.

However, our results need to be interpreted with caution due to the limited sample size of the present study, and note further studies are required to validate this observation. No significant differences between HEU and HU infants were seen in the baseline level of the inflammatory factor CRP. This result is different from Prendergast et al. (38) who showed that at 6 weeks of age, HEUinfants had significantly higher CRP than HU infants. The difference in CRP results between Prendergast et al. studies and our study could be explained by the fact that, contrary to Prendergast et al. study, almost all HEU infants in our study were not breastfed, limiting the transfer of breastmilk associated HIV-antigen or pro-inflammatory factors. Indeed, Adair et al. (39) demonstrated that in exclusively breastfed infants born to HIV-infected mothers, maternal factors (inflammatory factors included) correlated to infants CRP levels. Another possible explanation would be the difference in studies’ sizes and power.

Our data indicate that a number of HEU infants have impaired TLR4 function. More than half of HEU infants did not respond to TLR4-agonist (LPS) stimulation. The mechanisms explaining or leading the observed TLR4 anergy are unknown. Further studies should be conducted to confirm our observation and uncover the mechanism involved. Environmental factors *in utero* have been shown to modulate early life immune responses in different ways (40). Without affirming that it is the case here, it has been demonstrated by Piccinini et al. (41) that fetal antiretroviral exposure induced a blockade of NF-κB activation and degradation of its inhibitor IκBα. Furthermore, Equils and colleagues demonstrated that HIV-1 protease inhibitors block TLR4-induced NF-κB activation as well as LPS-induced IL-6 promoter transactivation (42). It might be possible that fetal exposure to other antiretroviral drugs has the same effect.

Accumulated data on HEU infants’ immune system suggest heterogeneity in their immune response. Some are able to mount strong responses to specific antigens; whereas, others showed a weak or absent to them (9–11, 28). It seems like any part or pathway of the immune system can be affected. Our study, although small, shows that selected HEU infants are characterized by a TLR4 anergy, whereas others respond to LPS, confirming the heterogeneity of HEU infants’ immune response. HEU infants enhanced response to PAMPs in their early life have also been reported by Reikie et al. (43).

Our findings would suggest that selected HEU infants (those not responding to LPS stimulation) would be particularly susceptible to Gram-negative bacteria infections. However, none of the HEU infants investigated in this study had severe infections in the year after their birth suggesting an alternative or compensatory mechanism to TLR4 function in infants non-responsive to LPS stimulation. It could also be that infants were well cared for after counseling of their mothers by our health-care workers.

In conclusion, HEU infants are characterized by a relative enhanced complement C3 expression and an impaired TLR4 response. TLR4 anergy can occur in HEU infants without necessarily translating to increased vulnerability to infection. The limited amount of whole blood obtained from enrolled infants made it difficult to further compare HEU and HU infants. This present pilot study needs to be validated with a larger population to confirm these observations (especially for complement C3) as larger your sample is, the more accurately it reflects the population.

## Ethics Statement

The National Laboratory of Public Health board approved the study. Parental consent was obtained for all infants.

## Author Contributions

JFDS is the principal investigators who conceived and designed the study. ACMS, OMN, and CNMM recruited participants and acquired the samples. ACMS, OMN, and AMN1 (Amandine) helped in designing the TLR4 function experiment. ACMS, OMN, and AMN1. did the experiments and all supervised by JFDS ML, RKB, and AMN2 (Armel) helped in recruited participants and acquired the samples; RKB and AMN2 did all the HIV testing. PNE helped writing the manuscript. JFDS did the analyses and wrote the manuscript with input from all authors supervised. All authors approved the final manuscript revisions.

## Conflict of Interest Statement

The authors declare that the research was conducted in the absence of any commercial or financial relationships that could be construed as a potential conflict of interest.
